# Understanding the influence of marine nutrients on insectivorous and herbivorous reptiles in the Gulf of California islands

**DOI:** 10.1371/journal.pone.0329414

**Published:** 2025-08-22

**Authors:** Ilse K. Barraza-Soltero, MCarmen Blázquez, Salvador Hernández-Vázquez, Antonio Delgado-Huertas, Víctor M. Muro-Torres

**Affiliations:** 1 Centro de Investigaciones Biológicas del Noroeste, S.C., La Paz, México; 2 Centro Universitario de la Costa Sur, Universidad de Guadalajara, Melaque, Jalisco, México; 3 Instituto Andaluz de Ciencias de la Tierra CSIC-UGR, Armilla, Granada, Spain; University of Hyogo, JAPAN

## Abstract

Marine subsidies in the extremely arid islands of the northern Gulf of California have been shown to be important enhancing primary productivity and fueling the terrestrial food webs. This effect has been proved in plants, insects, lizards and rodents. The aims of our study were first to determine whether insectivorous lizards from a wide array of islands, including some in the central and southern part of the Gulf, are consuming marine derived products, and secondly to assess its impact on herbivore lizards as well. We hypothesized that the availability and use of marine nutrients for lizards would vary depending on island aridity, island size, and the presence of seabird colonies nearby. To test the hypothesis, we analyzed the isotopic niches of 13 populations of insectivorous lizards (genus *Uta*) and 9 populations of herbivore iguanas (genera *Dipsosarus, Ctenosaura*, and *Sauromalus*) across 16 islands spanning 5 latitude degrees and 350 nautical miles. Our results showed that the proximity of seabird colonies play a key role in reinforcing the presence of marine origin nutrients in both insectivorous and herbivore populations. The ubiquity of seabird colonies on the northern islands combined with higher aridity in the northern part of the Gulf, creates a northward gradient in the importance of marine subsidies for both insectivorous and herbivore lizards, across the Gulf islands. δ^13^C variation in insectivorous lizards’ tissues was significantly correlated with island aridity/latitude, while the presence of significant seabird colonies nearby was significantly correlated with δ^15^N variation. The results were less clear in the herbivore group of species. Insectivorous and herbivorous share a large portion (70%) of their isotopic space. The incorporation of the highly enriched ^15^N to both type of lizards is happening for the two possible ways, the presence of guano, that may be driving plant fertilization, and the direct consumption of insects associated to the birds (or birds’ products, as eggs or corpses); but we could not differentiate between them. The size of the islands was not relevant in our results, possibly due to our sampling design. Additional isotopic analysis of plants and arthropods and in a gradient coast-inland could provide a more comprehensive view of the nutrient flow within these island ecosystems and the effect of the island size.

## Introduction

One of the main questions in behavioral ecology is understanding how species and populations adapt to resource fluctuations in changing environments across their distribution areas. Individual fitness is influenced by inter- or intra-specific interactions but also by climate, habitat heterogeneity, and access to available resources. Some low-productivity, isolated ecosystems, such as arid islands, present unique challenges for animal populations because of the limited habitat heterogeneity, general resource scarcity, and temporally unpredictable variation in resource availability [1–3]

Occasionally, some of those low-productivity systems are surrounded by richer environments such as forests or oceans that, being more productive, provide allochthonous subsidies into the low-productivity systems [4–7]. Examples include “sky islands” such as tepuis or high summits, as well as proper insular ecosystems such as small or arid oceanic islands. Allochthonous marine subsidies entering these arid islands facilitate interaction between oceanic and terrestrial ecosystems by transferring materials, thereby influencing the dynamics of recipient communities [8–10]. Arid islands, islets, and small rocks of the Gulf of California, with their low primary productivity (0–60 gm^-2^yr^-1^ dry mass) [11,12] and unique biodiversity [13], offer an ideal setting to study this phenomenon [14–17].

Marine subsidies, which are transported to terrestrial ecosystems through donor-controlled processes (e.g., deposition of macroalgae, detritus, and stranded organisms on shores) or animal-mediated mechanisms (e.g., consumption of marine invertebrates, guano deposition, and remnants from seabird reproductive colonies or roosting places) [8,16], can significantly impact recipient communities. On Gulf of California islands, these subsidies have been linked to increased populations of various species, including invertebrates, lizards, rodents, and plants [4,10,16–18].

Reptiles are the most common vertebrates inhabiting these islands. They have been used as models for exploring long-term isolation, life history, evolution, behavioral change, and expansion of trophic niches to include newly available marine resources [9,10,13,19,20]. The species there, some of them endemic, have the potential to develop plasticity in their feeding behaviors [13,20] and, in some cases, they exhibit convergent responses to environmental resource variations, regardless of phylogenetic relatedness [2,9].

Our study aimed to explore how lizards, whether herbivorous or insectivorous, utilize marine-derived nutrients across multiple islands in the Gulf of California. We hypothesized that the extent of marine components in lizard diets of the same genus would vary depending on factors such as island aridity [7], island size [21], and the presence of significant nearby seabird colonies [17,18]. To test this hypothesis, for insectivorous species we compared the isotopic niches of *Uta* lizard populations inhabiting the coastal fringes of several Gulf of California islands. For the herbivorous species we compared *Dipsosaurus*, *Ctenosaura* or *Sauromalus* iguana populations inhabiting different islands. This approach was deemed valid because geographic variation in the isotopic niche of generalist predators offers insights into spatial changes in their food webs [22], especially in basal resources fueling trophic webs [23]. We predicted that, despite potential individual variations, meaningful differences in species´ isotopic niches could still be detected across islands, reflecting variations in island aridity, island size, and seabird colony influence [24].

To assess and compare the trophic niches of insectivorous and herbivorous lizards, we employed stable isotope analysis of nitrogen (δ^15^N) and carbon (δ^13^C). Stable nitrogen isotopes are a powerful tool for tracing the flow of marine nutrients into terrestrial food webs [25]. Nitrogen isotope values (δ^15^N) increase predictably with each trophic level. Marine food webs, being generally longer than terrestrial ones, lead to higher δ^15^N values [26] in marine organisms, and marine-derived resources, such as stranded animals, exhibit elevated δ^15^N values in consequence. Also, marine algae have higher δ^15^N values than terrestrial plants. Usually studies consider a trophic discrimination factor (TDF) of approximately 3.4‰ [27,28], due to fractionation processes during protein synthesis and the excretion of lighter isotopes, although the factor could vary more than 9‰ withing a single species and tissue type. We used the generally ~3.4‰ because of the great variation in our samples (species, islands), and to make our results comparable with previously published data. One of the main sources for transferring marine-derived nitrogen onto the islands are seabird colonies. It can be directly through arthropods that feed on seabird by-products, such as faeces, eggs, corpses, and chicks, and hence they become good prey for their reptilian predators [10,29]. The other way is indirectly, because guano (which has a higher proportion of ^15^N compared to ^14^N due to the birds’ high trophic position and the preferential volatilization of the lighter isotope [30]) increases the nitrogen content in the soil, from where it is transferred to plant tissue and detritus, which then serves as a food source for detritivores and arthropods [17,18,31]. Variations in carbon isotope signatures (δ^13^C) between marine and terrestrial organisms also provide insights into long-term dietary patterns, particularly in species with mixed diets. Minimal carbon fractionation, around ~1‰ but also potentially highly variable [28], occurs during digestion and assimilation, resulting in consumer tissues reflecting the δ^13^C values of their food sources [25,32–34]. In this case, and for the same reasons that we considered in N TDF, we stayed with the traditionally used ~1‰ TDF for C. In terrestrial environments, plants exhibit a range of δ^13^C values depending on their photosynthetic pathways. C3 plants, the most common type, typically have very negative values (<−28‰) compared with C4 and CAM metabolism plants (−12‰ to −13‰) [33,35]. Marine phytoplankton and coastal algae have intermediate δ^13^C values (−12‰ to −24‰), while intertidal organisms feeding on algae can exhibit values similar to, or more enriched than C4 plants (−11‰ to −18‰) [10,36]. These distinctions allow for the differentiation of carbon sources incorporated into island-dwelling organisms, indicating whether they originated from marine or terrestrial environments [37].

Overall, we expected higher (less negative) δ^13^C and higher δ^15^N values in lizards living near seabird colonies [24], in lizards on smaller islands due to greater exposure to marine nutrients [21], and in lizards from more arid islands due to low primary productivity and the dominance of C4/CAM plants. Finally, we anticipated higher δ^15^N values in insectivorous lizards compared with herbivorous species, reflecting their higher trophic position.

## Materials and methods

### Study area

**Fig 1:**
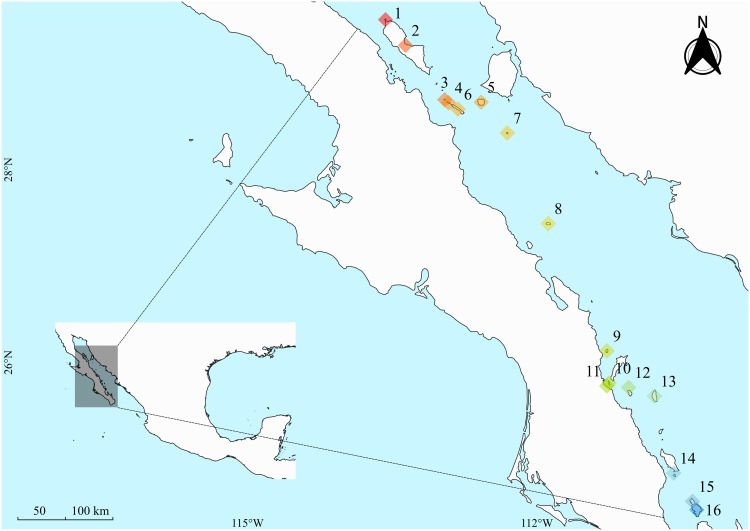
Sampled islands from the Gulf of California. The islands are identified with a color pattern following an aridity/latitudinal gradient where red/orange color means more aridity, yellow/green mid-aridity, and blue less aridity. 1: Mejia, 2. Angel de la Guarda, 3: Salsipuedes, 4: Las Animas, 5: San Esteban, 6: San Lorenzo, 7: San Pedro Martir, 8: Tortuga, 9: Coronados, 10: Danzante, 11: La Islita, 12: Las Galeras, 13: Santa Catalina, 14: El Pardito, 15: La Partida, and 16: Espiritu Santo. Map created using the open-source software QGIS [41]. We used two raster vector files from https://www.naturalearthdata.com/. “Land polygons including major islands” large scale data 1:10,000,000 and “Minor islands, islands that are 2 sq. km or less in size” large scale data 1:10,000,000. Derived from 1:250,000 World Vector Shoreline. The coordinates were taken from https://datos.abiertos.inecc.gob.mx/Datos_abiertos_INECC/CGACC/DocumentosRIslasMarias/Eje2_InstrumentosDelTerritorioInsularMexicano/CatalogoInsularDelTerritorioMexicano.pdf.

The Gulf of California hosts over 200 islands, islets, and small rocks, spanning 900 km across 7 degrees of latitude. Our study focused on 16 of these islands, covering 650 km and 5 degrees of latitude and encompassing a gradient of sizes, aridity levels, and presence of seabird colonies (Fig 1). Primary productivity on these islands fluctuates significantly across years because rainfall is unpredictable. There can be periods of three or more years without any rainfall. Rainfall events, when they occur, are typically concentrated within a few days, leading to ephemeral blooms of grass (C4 plants) [4]. The islands’ perennial vegetation, while generally scarce, is dominated by drought-tolerant Cactaceae and Agavaceae species, which use crassulacean acid metabolism (CAM). Other plant families present, including Leguminosae, Burseraceae, and Asteraceae, primarily use the C3 photosynthetic pathway [38,39].

CAM and C4 carbon fixation are the two photosynthetic pathways well-adapted to arid environments. CAM plants produce more sugar than C3 plants under conditions of high light and temperature, and can fix carbon while their stomata are closed, allowing for better water retention. C4 plants fix carbon during the day and are more efficient than C3 plants, keeping their stomata almost fully closed. However, C4 plants are less nutritious than C3 plants, containing less nitrogen and more fiber, and they are more difficult to digest [40]. The presence of each metabolic pathway can be identified through the carbon isotope analysis of the plant itself or its consumer.

### Model species and sampling

The Gulf of California islands host a unique lizard fauna, with many species endemic to one or more islands [13,42,43]. Our study focused on three insectivorous species of the genus *Uta*: *U. palmeri,* endemic to San Pedro Martir Island, *U. squamata,* endemic to Santa Catalina Island, and *U. stansburiana,* widely distributed across several islands. We also included four herbivorous species of the genus *Sauromalus* distributed across several islands: *S. hispidus, S. varius*, *S. slevini*, and *S. obesus* (Table 1). Additionally, we included *Ctenosaura conspicuosa,* an herbivorous species endemic to San Esteban Island, and two species of the genus *Dipsosaurus*: *D. dorsalis* and *D. catalinensis,* the latter being endemic to Santa Catalina Island. While both *Dipsosaurus* species are primarily herbivorous, they are known to opportunistically consume insects.

**Table 1:** Mean ± standard deviation isotopic values of tail-tissue samples of insectivorous and herbivorous lizards from different islands of the Gulf of California.

Island Species and individuals sampled (*n*)	Lizard diet	δ^13^C‰ ± SD	δ^15^N‰ ± SD	Island size (km^2^)	Island *AI*	Significant Seabird colonies
Mejia Island						
*Sauromalus hispidus* (9)	Herbivorous	−19.2 ± 3.38	31.55 ± 2.22	1.02	0.133	No
Angel de la Guarda Island						
*Uta stansburiana* (1)*	Insectivorous	−15.71	13.52	930.43	0.205	No
*S. hispidus* (1)*	Herbivorous	−22.96	2.01			
Salsipuedes Island						
*U. stansburiana* (8)	Insectivorous	−12.41 ± 2.50	23.73 ± 8.59	1.02	0.169	No
Las Animas Island						
*U. stansburiana* (7)	Insectivorous	−12.22 ± 1.81	28.38 ± 5.38	3.99	0.146	Yes
San Esteban Island						
*U. stansburiana* (4)*	Insectivorous	−13.44 ± 0.75	14.17 ± 0.82	39.66	0.184	No
*S. varius* (9)	Herbivorous	−17.1 ± 1.67	11.50 ± 1.71			
*Ctenosaura conspicuosa* (3)*	Herbivorous	−16.76 ± 1.73	10.74 ± 2.79			
San Lorenzo Island						
*U. stansburiana* (1)*	Insectivorous	−12.95	23.21	31.96	0.169	Yes
San Pedro Martir Island						
*U. palmeri* (4)*	Insectivorous	−13.42 ± 0.66	24.03 ± 2.28	2.71	0.223	Yes
Tortuga Island						
*U. stansburiana* (6)	Insectivorous	−11.78 ± 1.03	24.54 ± 4.27	11.30	0.233	Yes
Coronados Island						
*U. stansburiana* (6)	Insectivorous	−17.28 ± 2.36	18.42 ± 1.89	7.18	0.297	No
*S. slevini* (5)	Herbivorous	−21.12 ± 1.19	13.34 ± 4.99			
*Dipsosaurus dorsalis* (3)*^+^	Herbivorous	−23.70 ± 1.94	9.44 ± 0.97			
Danzante Island						
*U. stansburiana* (6)	Insectivorous	−18.00 ± 2.20	12.08 ± 1.55	4.15	0.320	No
*S. obesus* (2)*	Herbivorous	−18.38 ± 1.81	8.29 ± 0.99			
La Islita Island						
*D. dorsalis* (9) ^+^	Herbivorous	−17.1 ± 1.58	30.4 ± 1.65	0.02	0.304	Yes
Las Galeras Island						
*U. stansburiana* (6)	Insectivorous	−11.51 ± 1.22	38.22 ± 1.30	2.2	0.313	Yes
Santa Catalina Island						
*U. squamata* (1)*	Insectivorous	−18.71	14.48	38.89	0.377	No
*D. catalinensis* (1)*^+^	Herbivorous	−14.59	13.6			
El Pardito Island						
*U. stansburiana* (5)	Insectivorous	−13.27 ± 1.36	24.28 ± 1.55	0.01	0.336	No
La Partida Island						
*U. stansburiana* (5)	Insectivorous	−20.00 ± 2.51	10.68 ± 1.86	17.71	0.352	No
Espiritu Santo Island						
*U. stansburiana* (5)	Insectivorous	−18.23 ± 1.24	12.53 ± 1.18	83.78	0.375	No
*S. obesus* (2)*	Herbivorous	−21.23 ± 2.07	7.67 ± 4.22			

The sample size is detailed in parentheses. Island size (km^2^), island aridity index (*AI*), and presence/absence of significant seabird colonies are indicated for each island.* Individuals not included in the isotopic ellipses’ analyses per island. ^+^Not-strict.

We conducted diurnal surveys in all available habitats within 200 meters of the shoreline on each island. We did not collect the GPS data of each precise capture. Lizards were captured using a fishing rod with a thread loop at the end. For each island visit, we collected, when possible, two sets of samples (two populations) representing insectivorous and herbivorous lizards. After each capture, we trimmed approximately 1 cm of the tail tip and preserved the tissue samples in vials with 70% alcohol. This research was carried out under SEMARNAT permits SGPA/DGVS/ −10182/11–07594 7/15–2971/20–05480/21 for handing animals. No organisms were sacrificed for this work.

To characterize the isotopic baseline of primary producers, we collected and analyzed plant samples from Angel de la Guarda Island. This included C3 plants (*Larrea tridentata, Datura spp., Euphorbia lomelii, Marina spp, Batis maritima, Lupinus spp*. *Cucurbita cordata*), C4/CAM plants (*Stenocereus gummosus, Cylindropuntia cholla, C. imbricata, Pachycereus pringlei, Lophocereus schottii, Stenocereus thurberi, Mammillaria dioica, Opuntia fulgida, Agave spp., Echinocactus platycathus*), and marine seaweed (species not determined). We compared the average isotopic values of these plants to the isotopic space occupied by herbivorous lizards. This plant data was complemented with values from the literature [4,10,16,17,23,44] (S1 Table). While we did not collect arthropod samples for direct isotopic analysis, we incorporated data from the literature [4,10,16] on arthropod isotopic signatures from the region to provide context for insectivorous lizard isotopic values (S2 Table).

Fieldwork was conducted between November 2013 and September 2021 (S3 Table). This period included two El Niño events (September 2014 – May 2016), (July 2018 – June 2019) and three La Niña events (July 2016 – January 2017; September 2017 – May 2018; July 2020 – September 2021) [45]. To conduct an exploratory analysis of the effect of those events on our samples, and because of the effect of the ENSO events on the seabird colonies, and on the island vegetation, we assigned a category to each population depending on whether the samples had been taken during neutral season or a period covered by an El Niño or La Niña event (S3 Table) and we then compared the values of δ^13^C‰ and δ^15^N‰ of each group using a non-parametric Kruskal-Wallis test. Only populations of *U.stansburiana* had different average values of δ^15^N‰ across neutral and El Niño years (K-W chi-squared = 6.226, df = 2, p-value = 0.04; neutral n = 11, average δ^15^N‰ = 25.3 ± 4.41; Niño n = 35, average δ^15^N‰ = 19.6 ± 7.86).

### Stable isotopes analysis

All stable isotope analyses were carried out at the Stable Isotope Laboratory of the Instituto Andaluz de Ciencias de la Tierra (CSIC-UGR, Granada, Spain). Homogenized lizard tail tips were analyzed for carbon and nitrogen isotopic composition using a Carlo Elba NC1500 elemental analyzer (Milan, Italy) with a Delta Plus XP (ThermoQuest, Bremen, Germany) mass spectrometer (EA-IRMS).

Lipid extraction was not performed, given that lizard tail tips typically exhibit low lipid content [23,46]. This approach is valid when the lipid content is under 10%, indicated by a C:N ratio value around 4 [47], and only one of our samples per group had a C:N ratio > 5 (6.8) (S3 Table). Stable isotope values are presented as δ values per mil, calculated using the formula; δ = (*Rsample/Rstandard* – 1) * 1000, where *R* represents the ratio of heavy to light isotopes (^13^C/^12^C for δ^13^C and ^15^N/^14^N for δ^15^N).

Commercial CO_2_ and N_2_ served as internal standards for carbon and nitrogen analyses, respectively. For carbon, we used two internal standards of −30.63‰ and −11.65‰ (Vienna Pee Dee Belemnite; V-PDB), analyzed every ten samples. For nitrogen, we used two internal standards of −1.02‰ and +16.01‰ (AIR). The calculated precision, after correction for daily mass spectrometer drift, based on standards systematically analyzed across analytical batches was better than ±0.1‰ for both δ^13^C and δ^15^N.

### Isotopic niche metrics

We analyzed isotopic niches for insectivorous and herbivorous lizard populations separately. For each island population with at least five samples, we estimated isotopic niches using Bayesian Analysis of Standard Ellipses with the SIBER package in R [48,49]. We calculated the Standard Ellipse area (SEAc) with 95% of the data. Additionally, to obtain basic summary statistics of each population, we calculated Layman metrics [50]. They include total area of the convex hull with 100% of the data (TA), Carbon range (CR), Nitrogen range (NR), mean distance to centroid (CD), and mean nearest neighbor distance (MNND). These measures allow us to compare the populations.

To evaluate the impact of seabird colonies on the insectivorous, we grouped the 65 tail-tip samples into two categories: islands with significant seabird presence and islands without, disregarding aridity/latitude and island size. We then calculated isotopic niche metrics for each group

We also used the “Ellipse Overlap” function within SIBER to quantify shared isotopic space between island populations and between insectivores and herbivores.

### Statistical analysis

To assess aridity and latitude relationships among the islands, we calculated the aridity index (*AI*) for each island using data from the Mexican Insular Territory layer [51] and WorldClim bioclimatic variables: Annual Precipitation and Mean Annual Temperature spanning the period of 1970−2000 [52]. We used these data because specific climate data for the Gulf of California islands were unavailable. Despite the existence of numerous aridity indices, we chose the Köppen aridity index for its recognized accuracy in reflecting arid climates in North America [53,54], and because it collects the rainfall data for long periods, which would include Enso events, that we noticed could be important for the δ^15^N levels of insectivorous populations (see model species and sampling). The data were processed using the Geographic Information System software QGIS 3.16.13 [41], applying Köppen’s formula for aridity: *AI*_*Köppen =*_ *MAP/ MAT + 33,* where *MAP* denotes Mean Annual Precipitation, *MAT* denotes Mean Annual Temperature, and 33 is a constant. *AI* values equal to or greater than 0.32 indicate less arid conditions, values between 0.21–0.31 indicate moderate aridity, and values at or below 0.20 denote hyper-aridity. Subsequently, we explored correlations between latitude, *MAT*, *MAP*, and *AI* using Spearman correlation.

Seabird colony presence was determined based on the literature [2,10,17,18,29] and on field observations over the years. Because it has been proved that the impact of the colony’s decays spatially and temporary [24], we categorized its presence as positive only when colonies (nests or perches) were evident on at least 25% of the island (for which we made a coastal survey by boat) or were close (less than 1 km) to our sampling area; otherwise, any seabird colony presence was categorized as “absence”. While we recognize the significant effects of guano on soil fertility and biogeochemistry, we lacked information on historical seabird colonies, except for very evident and current colonies on Isla Rasa, San Lorenzo and San Pedro Martir which we considered as with birds [55–58]. Given this limitation and the short lifespan of lizards, we excluded historical seabird data from our analysis.

We used Linear Mixed-Effects Models (LME) with response variables (*y*) being carbon and nitrogen values and independent variables being aridity index, island size, and the presence/absence of significant seabird colonies. We did not have the precise location of each lizard, and because we visited three of the islands more than once (S3 Table), we included the island as a random effect in order to avoid pseudoreplication (δ^13^C or δ^15^N ~ aridity index+ island size + presence/absence of significant seabird colonies, random = ~ 1 | island ID). Separate models were used for insectivorous lizards (n = 65) and herbivorous lizards (n = 44). All analyses were performed in R version 4.4.2 (2024-10-31) -- “Pile of Leaves”, using the package “nlme” with the “lme” function [59].

## Results

We found significant negative correlations between Latitude and Mean Annual Temperature (*MAT*) (Spearman r = −0.62, p = 2.6e^-09^), Mean Annual Precipitation (*MAP*) (Spearman, r = −0.95, p < 2.2e^-16^), and the aridity index (*AI*) (Spearman, r = −0.95, p < 2.6e^-16^). These results indicate that northern islands in the Gulf of California tend to be cooler, drier, and more arid than the southern islands.

### Insectivorous lizards

We analyzed 65 tail-tip samples from insectivorous lizards (Table 1). As predicted, lizards on more arid islands rely more on marine-derived nutrients and had significantly less negative δ^13^C, while on islands with presence of sea birds’ colonies, we found lizard tissues containing significantly higher values of δ^15^N (Table 2). The size of the islands was not significantly correlated with the isotopic variation in the lizard´s tissues. The correlation between the variables was similar in both models C and N (aridity/size correlation 0.24, 0.26; sea birds presence/aridity correlation 0.46, 0.47 and presence of seabirds/size correlation 0.28, 0.30).

**Table 2:** Coefficients of the Linear Mixed-Effects Model for the variation of δ^13^C and δ^15^N in insectivorous lizards.

	Estimate	SE	F	*T*	*df*	*P*
LME δ^13^C or δ^15^N ~ aridity index+ island size + presence/absence of significant seabird colonies, random = ~ 1 | island ID						
**δ^13^C**						
AIC = 294.4436 BIC = 307.1021, loglik = −141.2184						
Island	1.849					
**Fixed effects**						
Intercept	−10.69	2.53	697.94	− 4.21	51	–
*Aridity Index*	−18.691	8.11	*12.017*	−2.30	10	0.044*
Island Size	−0.001	0.003	1.13	−0.48	10	0.63
Bird’s colony (presence)	2.51	1.34	3.50	1.87	10	0.0908
**δ^15^N**						
AIC = 400.112 BIC = 412.777, loglik = −194.052						
Island	5.64					
**Fixed effects**						
Intercept	17.76	7.45	156.09	2.38	51	–
Aridity Index	−3.90	23.76	2.259	−0.16	10	0.872
Island Size	−0.004	0.008	2.259	−0.573	10	0.579
*Bird’s colony (presence)*	*11.12*	*3.95*	*7.886*	*2.81*	*10*	0.0185*

**Fig 2:**
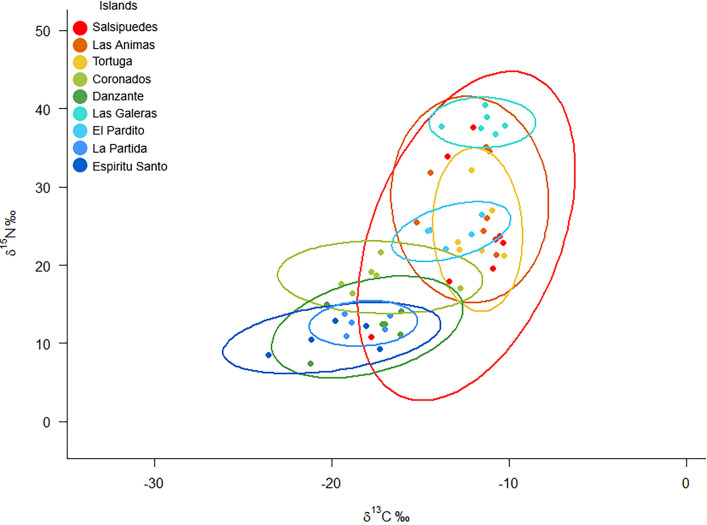
Bayesian ellipses representing the isotopic niche of *Uta stansburiana* on the islands. The color pattern of each island corresponds to the latitudinal gradient shown in Fig 1.

The isotopic spaces of *Uta stansburiana* populations generally followed a latitudinal (aridity) gradient with more influence of marine nutrients on the northern (more arid) islands with two exceptions (Fig 2). First, the population from El Pardito Island showed less negative isotopic C (−13.27 ± 1.3‰) than other islands at its latitude. Second, the population from Las Galeras Island in the middle of the Gulf exhibited the least negative δ^13^C values (−11.51 ± 1.22‰) of all.

The lizards from these two islands have also high δ^15^N values (24.28 ± 1.55‰, 38.22 ± 1.30‰) indicating strong influence of marine nutrients. El Pardito is tiny and the only habited island on the Gulf, the fishermen living there leave fish entrails quite regularly on the shoreline. Las Galeras support a large yellow-footed seagull colony (*Larus livens*) present across almost the entire flat part of the island, where the lizards live and forage (personal obs) [60] and its vegetation is comprised only of cactus (CAM).

*Uta* populations living close to seabird colonies on islands of the northern part of the Gulf also had elevated δ^15^N values (Table 1, Fig 1). Salsipuedes’ population (northern non-bird island) also had elevated δ^15^N values.

The carbon range value (CR) was wide on the population from Salsipuedes Island, and on the population from Coronados in the middle Gulf, and narrower on the islands with sea bird colonies. The minimum range corresponded to the less arid sampled island, Espiritu Santo (Table 3), on the southern part of the Gulf.

**Table 3:** Stable isotope niche metrics for insectivorous lizards.

	Salsipuedes	Las Animas	Tortuga	Coronados	Danzante	Las Galeras	El Pardito	La Partida	Espiritu Santo
TA	82.80	39.82	15.59	18.22	23.75	6.56	5.81	14.71	5.35
SEAB	61.78	30.74	13.81	14.01	17.10	5.04	5.99	13.51	4.61
CR	2.09	0.42	0.37	1.80	0.32	0.50	0.31	0.64	0.20
NR	5.64	8.10	5.02	2.08	1.54	1.62	1.92	1.11	1.93
CD	3.00	4.05	2.51	1.37	0.78	0.85	0.97	0.64	0.97
MNND	6.01	8.11	5.03	2.75	1.57	1.70	1.95	1.28	1.94

TA = Total Area, SEAB = Bayesian Standard Ellipse Area, CR = Carbon Range, NR = Nitrogen Range, CD = Mean Distance to Centroid, and MNND = Mean Nearest Neighbor Distance. Shading area, islands with seabird colonies.

Las Animas and Salsipuedes, on the north, and Tortuga in the middle of the Gulf, had the widest nitrogen range values (NR) (Table 3). Individuals from these populations had also disperse position within the isotopic space (MNND) suggesting variability in their diets. Populations from La Partida and Danzante Islands, had the narrowest nitrogen range values, suggesting less isotopic diversity in their diets.

### Importance of seabird colonies

**Fig 3:**
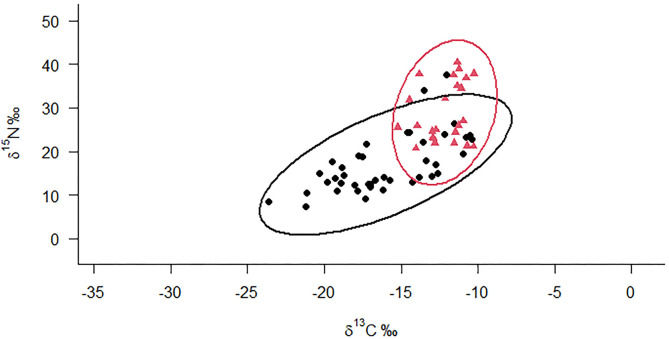
Bayesian ellipses show isotopic niches of *Uta* lizards on islands with and without significant seabird colonies. Red triangles represent the population of lizards living in islands with presence of significant seabird colonies and black circles represent populations living on islands without significant seabird colonies.

As expected, overall insectivorous lizards from islands with seabird colonies showed less negative δ^13^C and higher δ^15^N values compared with insectivorous lizards from islands without seabird colonies (Fig 3). Lizards from islands with seabird colonies occupied smaller areas in the isotopic space (smaller TA and SEABc), and had narrower carbon range (CR values) (Table 4). The NR was quite similar and also MNND, indicating the same dispersion in the isotopic space and from the centroid (CD). Ellipse overlap analysis revealed that the two groups shared 50.33% of the isotopic space, suggesting considerable overlap in dietary sources.

**Table 4:** Stable isotope niche metrics for insectivorous lizards from islands with presence or absence of significant seabird colonies.

	TA	SEAB	CR	NR	CD	MNND	*n*
Presence	76.77	29.95	0.97	8.15	4.10	8.21	24
Absence	168.23	53.81	3.28	7.60	4.14	8.28	41

TA = Total Area, SEAB = Bayesian Standard Ellipse Area, CR = Carbon Range, NR = Nitrogen Range, CD = Mean Distance to Centroid, MNND = Mean Nearest Neighbor Distance, and n = sample size.

### Herbivorous lizards

We analyzed 44 tail-tip samples from iguanas of the genera *Sauromalus*, *Ctenosaura,* and *Dipsosaurus* (Table 1). We did not find an effect of aridity, the presence of seabird colonies or islands size on the isotopic variation of the tail tissues on the herbivorous lizards either in C or N (Table 5). The correlation between the variables was similar in both models C and N (aridity/size correlation 0.16, 0.23; sea birds presence/aridity correlation −0.15, −0.10 and seabirds presence/size correlation 0.13, 0.14).

**Table 5:** Coefficients of the Linear Mixed-Effects Model for the variation of δ^13^C and δ^15^N in herbivorous lizards.

	Estimate	SE	F	*T*	*df*	*P*
LME δ^13^C or δ^15^N ~ aridity index+ island size + presence/absence of significant seabird colonies, random = ~ 1 | island ID						
**δ^13^C**						
AIC = 213.75 BIC = 223.885, loglik = −100.876						
Island	2.49					
**Fixed effects**						
Intercept	−18.60	3.47	365.4750	− 5.35	36	–
Aridity Index	−1.10	12.21	0.0602	−0.09	4	0.8183
Island Size	−0.004	0.003	1.4575	−1.10	4	0.294
Bird’s colony (presence)	1.83	2.89	0.4018	0.63	4	0.5606
δ^15^N						
AIC = 233.61, BIC = 243.75, loglik = −110.80						
Island	6.70					
**Fixed effects**						
Intercept	31.81	8.84	37.1801	3.59	36	–
Aridity Index	−61.53	30.04	1.5095	−2.04	4	0.2866
Island Size	−0.01	0.008	6.1443	−2.12	4	0.0683
Bird’s colony (presence)	17.29	7.39	5.4628	2.33	4	0.0796

Although for herbivorous lizards we did not detect an effect of the aridity on the whole, we noticed some differences between populations. For instance, it seems more a prevalence of CAM/C4 plants in the diet *Sauromalus* from Mejia Island (δ^13^C average −19.2 ‰), and *Ctenosaura* and *Sauromalus* iguanas from arid San Esteban Island`s (with δ^13^C average of −16.76, and −17.1‰), compared to a diet more towards C3 plants on iguanas from less arid Coronados Island’s, *Dipsosarus* and *Sauromalus* (δ^13^C average of −23.70 and −21‰ respectively) or Espiritu Santo *Sauromalus* (δ^13^C average of −21.23‰). Individual *Sauromalus* iguanas from Mejia and San Esteban should have more variation in their diets because they showed broader carbon range values (CR) than *Sauromalus* from Coronados (Table 6).

**Table 6:** Stable isotope niche metrics for herbivorous lizards.

	Mejia/ *S. hispidus*	San Esteban/ *S. varius*	Coronados/ *S. slevini*	La Islita/ *D. dorsalis*
TA	37.14	10.83	12.94	9.46
SEAB	27.03	6.88	16.2	6.94
CR	2.06	2.76	0.94	0.06
NR	0.93	2.85	4.62	0.22
CD	1.13	1.98	2.35	0.11
MNND	2.26	3.97	4.71	0.23

TA = Total Area, SEAB = Bayesian Standard Ellipse Area, CR = Carbon Range, NR = Nitrogen Range, CD = Mean Distance to Centroid, and MNND = Mean Nearest Neighbor Distance.

There are two *Dipsosaurus* populations with less negative values of δ^13^C from less arid islands. One is the population from the inlet, la Islita, very close to Coronados, (δ^13^C values −17.1 ‰) and with the same aridity index but almost barren. They were probably feeding on the few Cactaceae (CAM/C4 plants) present there. The other, is a single individual of *D. catalinensis* endemic to Santa Catalina Island. Its tissues had levels δ^13^C corresponding also to a diet based on C4/CAM plants (−14.59‰).

Our results also highlight the importance of the sea birds’ colonies for the iguanas to be able to incorporate marine nutrients into their tissues. For example, la Islita was the only island sampled for herbivores with a large sea bird colony, in this case of yellow-footed seagull (*L. livens*), and some nests of blue heron *A. herodias* [60]. *Dipsosaurus* there had very high levels of δ^15^N (average = 30.4‰). We found also strong marine influence on the diet of Mejia Island’s *Sauromalus* individuals (δ^15^N average = 31.55‰). Here our results are probably due to the fishermen activity on the shores of this island. On the other side *Sauromalus* from Espiritu Santo, with no marine influence had lower δ^15^N levels (average = 7.67‰).

We only collected one individual of *S. hispidus* from Angel de la Guarda Island, found perched on a *Pachycormus discolor* tree (C3 plant). Its δ^13^C and δ^15^N values were closer to those of the iguanas from the southern islands. Angel de la Guarda is a massive island with scarce vegetation; its aridity index is higher than the southern islands, and lower than the smaller islands on the north, but this value has in any event to be treated with care because it was only one individual feeding on a C3 plant.

The isotope niche metrics indicated that the *Dipsosaurus dorsalis* population from La Islita Island had a diet with minimal isotopic variation, exhibiting the narrowest carbon (CR) and nitrogen range (NR) values of all the iguanas along with small distance to centroide and individuals packed on the isotopic space (Table 6).

*S. hispidus* from Mejia had narrower NR than *S. varius* from San Esteban and both less broad than *S. slevini* from Coronados Island in the south. Also, the individuals from Coronados and San Esteban, were more disperse on the isotopic space than *Sauromalus* from Mejia (Table 6). The small CR in Coronados *Sauromalus* along with its lower δ^15^N values suggest a diet more focused on C3 plants.

### Isotopic niche of insectivorous vs herbivorous lizards

Insectivorous and herbivorous lizards share a wide (0.694) overlap in isotopic niches (Fig 5). Insectivorous lizards displayed less negative values for C and higher values for N, consistent with their higher position in the trophic web.

**Fig 4:**
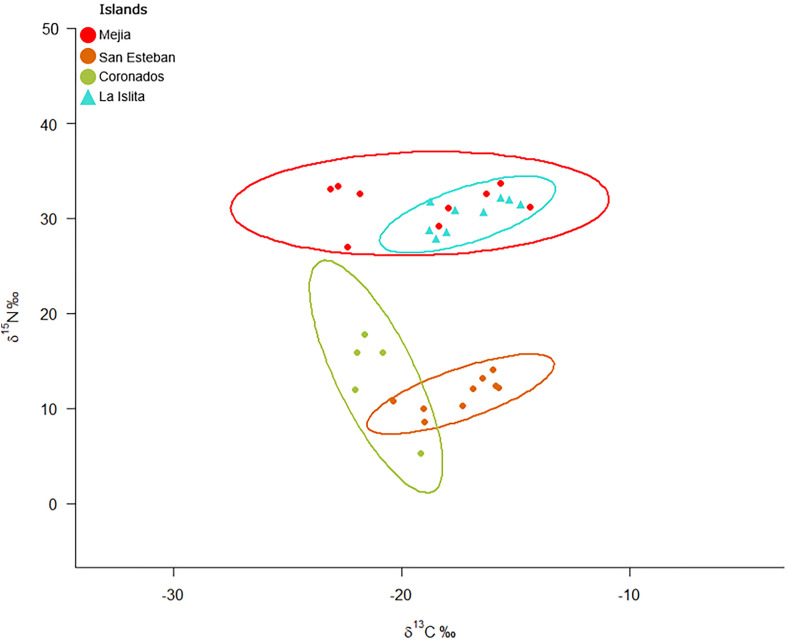
Bayesian ellipses of the isotopic niche of herbivorous iguanas on the islands. The color pattern of each island corresponds to the latitudinal gradient shown in Fig 1. Circles represent species of the *Sauromalus* genus, while triangles represent *Dipsosaurus dorsalis*.

**Fig 5:**
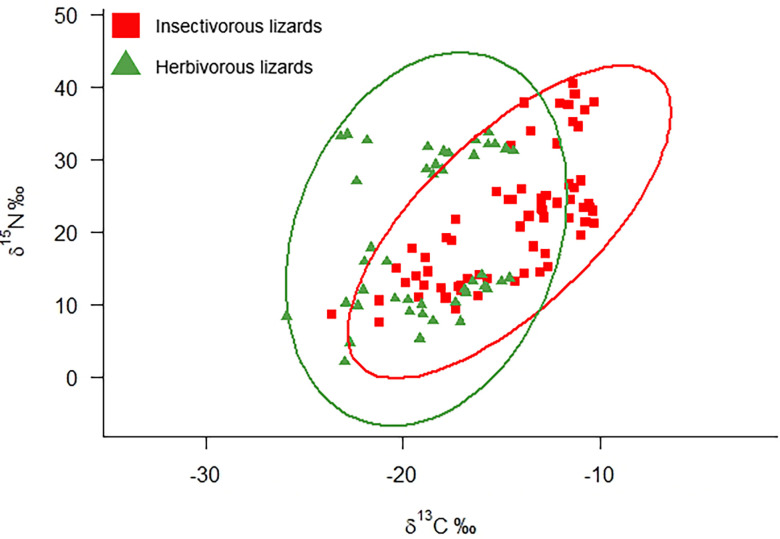
Isotopic niche of insectivorous and herbivorous lizards from the Gulf of California Islands. Insectivorous lizards are denoted by red squares and herbivorous lizards by green triangles.

Analysis of isotopic niche metrics revealed that insectivorous lizards had a carbon range value almost twice that of herbivorous lizards. The NR was similar but herbivorous lizards, had a larger TA and Standard Ellipse Area. This larger ellipse resulted from the two groups with different nitrogen values from islands with and without seabird colonies (Fig 4). Both groups exhibited similar diversity and packing metrics (Table 7).

**Table 7:** Average ± Standard deviation of isotopic values and niche metrics of insectivorous and herbivorous lizards.

	δ^13^C‰ + SD	δ^15^N‰ + SD	TA	SEAB	CR	NR	CD	MNND	*n*
Insectivorous	−14.6 ± 3.35	21.38 ± 8.81	214.33	66.82	4.66	12.32	6.59	13.18	65
Herbivorous	−18.8 ± 2.91	18.99 ± 10.52	276.75	95.49	2.13	14.33	7.24	14.49	44

TA = Total Area, SEAB = Bayesian Standard Ellipse Area, CR = Carbon Range, NR = Nitrogen Range, CD = Mean Distance to Centroid, MNND = Mean Nearest Neighbor Distance, and *n *= sample size

## Discussion

Since the pioneering studies conducted by the Polis group in the 1900s, the importance of allochthonous subsidies to the arid islands of the northern part of the Gulf of California has been well documented, particularly for insect, spider, and rodent populations [5–8]. Posterior research has further confirmed that insectivorous lizard populations, as well as cardon forests and other vegetation, also rely heavily on these marine subsidies, especially those derived from guano deposits [10,12,17]. The primary objective of our study was to assess whether the importance of the marine subsidies extends to all the islands, including those less arid in the southern region, and to evaluate their impact on herbivores lizards as well. Our first result was that insectivorous lizards from all the sampled islands were able to incorporate marine-derived nutrients into their diets.

The δ^13^C variation in each population was significantly associated with the degree of aridity of the islands where they live. The carbon (δ ^13^C) values were less negative on the northern (more arid) islands and overall, the δ^13^C values recorded for *Uta* lizards in this study (−11.78 to −20.00‰) are similar to those reported for the same species on the northern Gulf of California islands (−13.09 to −18.99‰) [10]. Both values are consistent with a diet based on insects feeding on C4/ CAM plants. When insectivorous lizards are consuming insects that feed on Cactaceae or other C4/ CAM metabolism plants, and because the low TFD, their tissues will reflect the C signature of the insects and the plant [23,61]. That could mean that the aridity gradient on δ ^13^C values is reflecting a prevalence of this type of vegetation towards the northern islands. But it is interesting that the δ^13^C values in our study remain less negative than those found for *Uta stansburiana* in the Chihuahuan Desert (−19 to −22), a highly arid region at a similar latitude but subject to entirely continental conditions [40]. Also, lizards from Atacama Desert, in the southern hemisphere, lizards from Namibia Desert, or insectivorous skins from Australian desert have more negative δ^13^C values that our insular *Uta* lizards although they feed on C4/CAM plants [62–64]. We might then suppose it must be additional marine elements in the diet of the insular lizards besides the prevalence of C4/CAM plants. It has been demonstrated on the Upper Gulf islands, that *Uta* lizards are significantly more abundant on the shorelines of the islands than in the interior, regardless the size of the island or if the islands have sea birds’ colonies or not [10]. The coastal fringe can support higher numbers of lizards because it has more food coming from the sea (insects, amphipods or crustaceans feeding on algae or in stranded carrion, like in Rasa Island where *Uta* feeding grounds are the drying sand flats at low tide, obs. pers). The authors report that aprox 40% of the coastal *Uta* lizards’ food contains arthropods who have been feeding on algae [10]. This is remarkable, because our lizards came precisely from the coast of the islands, and that may be the additional element for our samples to have less negative δ^13^C than others in continental deserts. In other island lizards, from temperate regions like Azores [65] or in coastal areas like South Puget Sound on the Pacific Coast of North America, [66] it has been probed that there is indeed an influence of marine nutrients in lizards diets on the shore that decrease towards lizards living inside the island or the forest. This was evident on the *Uta* numbers registered by [10] on the very arid and very poor productive northern islands of the sea of Cortes, but only in terms of abundance. They were more than four times abundant on the coast in the islands with sea bird colonies and until nine more times abundant in islands without sea bird colonies. But it will be interesting to test it in terms of isotopic δ^13^C and δ^15^N values.

The range of δ^13^C was slightly broader on the arid islands of the north, and was narrower on lizards from sea bird islands. It was minimum on the southern island of Espiritu Santo (CR = 0.2). On Salsipuedes, an arid northern island without seabirds, we found the population spread out in the isotopic space with wide ranges or C and N (Table 3). It could reflect some individuals feeding on the insects living of shore debris, and others feeding on insects feeding on plants.

The δ^15^N in insectivorous as expected, was significantly higher on populations close to seabird colonies, despite the aridity/latitude or the size of the island. Previous studies have highlighted the importance of seabirds in incorporating marine nutrients into terrestrial food webs, primarily through guano which enriches the soil with nitrogen [10]. Studies of seabird-island soils report δ ^15^N values ranging from 28.26 to 35.64‰ [12,17,18,36]. This nitrogen enrichment enhances plant productivity and rises up the food web, supporting populations of animals consuming detritus, plants, and seeds, [17,18,29,31]. Additionally, seabird colonies themselves provide a good habitat for arthropods and other scavengers feeding on seabird by-products, such as faeces, feathers, eggs, and carcasses, all of which insectivorous lizards may consume [6,10,16,29,67]. Although we could not distinguish between these two sources, our results strongly support the importance of seabird colonies as a primary marine nutrient source for arthropod populations [4,10,16] and insectivorous lizards along the entire range of the Gulf islands (S2 Table, S1 Fig), mainly regarding nitrogen isotopes. Areas near seabird colonies may act as nutrient-rich hotspots, offering a higher availability of insects [24], which likely leads to more generalized lizard diets, as observed in *Uta palmeri* from San Pedro Mártir Island [2]. We have commonly observed insectivorous lizards (e.g., *Uta*, *Urosaurus*, or *Aspidoscelis*) around the nests of terns, seagulls, boobies, or brown pelicans, even outside the birds’ breeding season. This may be because these nests, even when empty, offer shade and protection. Another example is *U. stansburiana* lizards on Las Galeras Island, they have year-round access to guano-derived nutrients, which results in high δ^15^N values.

The nitrogen range on the tissues of our insectivorous lizard samples (10.68 to 38.22‰) exceeds those reported previously for the northern islands [10], probably because our study included samples from southern islands as well, where some arthropods might feed on plants growing on less subsidized ground, although lizards from islands with or without sea bird colonies shared 50% of the isotopic space, meaning that the marine nutrients are present in both south and northern islands.

So, the presence of seabird`s colonies and the aridity index, which correlates with the south-north latitudinal gradient, were the two factors contributing to the presence of marine nutrients in the tissues of coastal *Uta stansburiana* lizards, and not the size of the islands along the Gulf of California.

Rainfall on these islands is generally scarce, with some years experiencing no rain at all. When rainfall does occur, it is typically associated with late summer (August-October) Pacific hurricanes in the southern and central Gulf or with cooler months (November- February) on the Upper Gulf islands [19,45]. However, these patterns can vary during El Niño/La Niña events. Although neither the frequency nor the intensity of hurricanes is clearly associated with El Niño/La Niña, El Niño years tend to show a mild positive rainfall anomaly along the southern Gulf coast of the Baja California Peninsula [68]. In the northern islands, during dry years, arthropod composition primarily relies on kelp, carrion, and parasitic flies [69] with presumably δ ^13^ C values less negative and δ ^15^N high values. After rainfall, there is a pulse of C4 plant production and seasonal growth of C3 plants [40], leading to an increase in herbivorous arthropods that consume both C3 and C4 plants [7] (more negative δ ^13^ C values). Rainfall also mobilizes nutrients and increases guano availability, making nitrogen (^15^N) more available.

During El Niño events, however, rising sea surface temperatures deplete the surface waters of plankton and fish. As a result, seabird breeding colonies typically decline, or may not occur at all on certain islands in those years [55,70]. Our data partially support this pattern as *Uta* samples from El Niño years had lower δ ^15^N values, but no differences in δ ^13^C values. Additionally, alongside this complex scenario, failures in nesting on traditional rookeries have been linked to global warming and overfishing in the Gulf [55–58]. Given these uncertainties, within the scope of this study we could not make predictions regarding El Niño/La Niña events. We focused instead on the aridity index, which accounts for total annual precipitation over long periods including both El Niño and La Niña years.

For herbivorous lizards any of the tested variables had significant effect on the isotopic variation. The δ^13^C values recorded for iguanas in this study (−16.76 to −23.70‰) were more negative that in insectivorous while δ ^15^N values (2.01 to 31.55‰), were not very different. While the importance of marine subsidies for herbivores on these islands has not been previously studied, the diet of giant *Sauromalus* (*S. varius* and *S. hipidus*) was studied on 1988 in several islands, including Mejia and San Esteban [71]. According to this study the chuckwallas are quite selective, choosing only 24% of the flora species of the islands, preferring shrubs as a food-base through the year when the annuals are not available, and choosing very actively forbs when appeared; cactus representing only 7% of their diet. In total they feed on 42 species of plants from 25 families. *S. hispidus* in Mejia (δ^13^C values −19.2‰), chooses ten species, it prefers flowers and leaves of *Perytile emoryi*, a forb with C3 metabolism, when available in the summer, and *Amaranthus palmeri* (C4) (leaves and seeds) in autumn and spring; The only registered Cactaceae (C4) was *Opuntia fulgida* in the autumn. The diet of *S. varius* endemic to San Esteban (δ^13^C values −17.1) included 22 species from 12 families, the majority of them C3, although cactus (C4) fruits and flowers represented 13%. On iguanas from these northern arid islands CR is wider (and more negative δ^13^C values), that on less arid southern Coronados or Espiritu Santo, these results suggest that they might prefer C3 plants when available, because generally C3 plants are more nutritive and easier to digest than C4/CAM, [40]. But we consider the results of this study only a first approach because there are many factors that could be important in the isotopic values, i.e., in a recent study in land iguanas on Galapagos [61] authors find isotopic niche differences between sympatric species and sexes due to their choice of food, mediated by their body size, and the productivity (NDVI) of their microhabitat.

The presence of significant seabird colonies directly increases nitrogen values in the soil, which are then reflected in the δ^15^N values of the plants and on ultimacy on the iguanas’ tissues (S1 Table, S2 Fig). Old, mineralized guano likely serves as a primary agent fertilizing the soil and plants, enriching isotopic signatures across the ecosystem [17,18,72,73], C3 and C4 plants on bird islands had significantly greater δ^15^N values than plants with the same photosynthetic pathway in both coastal and inland habitats [10]. Additionally, in the northern islands, characterized by extreme aridity, previous studies have shown that allochthonous nutrients support primary productivity and plant growth rates [5,8,17,18,74]. Thus, even in areas with sparse vegetation, the plants consumed by iguanas are likely to be enriched with marine nutrients, particularly nitrogen, this point has been demonstrated in Caribbean iguanas. There subsidized island support more abundant populations and also of bigger size animals [75]. The populations from southern islands with no seabird colonies showed lower δ^15^N values. The only island where we sampled iguanas and hosted an important sea bird colony is La Islita. There *Dipsosaurus dorsalis* was very abundant and its diet exhibited high δ^15^N values, the lowest packing metric values, and values of δ^13^C around −17‰, consistent with CAM metabolism of the Cactaceae living on the island. *S hispidus* from Mejia had also unexpectedly high δ^15^N values (31.55‰), much higher than those at the top of the food chain in Baja California sharks, where δ^15^N values range from +12.3‰ to +18 ‰ [76] and are comparable to la Islita's *Dipsosaurus.* Despite Mejia is not hosting big seabird colonies, organic matter mineralization on the island may be causing ^15^N enrichment in plants. Several factors may be behind this mineralization as fishermen activity leaving carcass on the shore (pers. observation), or other elements like, i.e., ocean spray [77].

The lack of island size effect on the isotopic values of lizards was surprising, given the range of island sizes in our study. Small islands have larger coastline perimeters, offering more shoreline area accessible for lizards, and some studies have suggested that ocean spray might influence terrestrial nutrients, and lizards from coastal areas had significantly greater δ^15^N values relative to populations in unsubsidized areas [10]. However, our sampling design, which collected samples approximately 100–200 m from the shore, may have overlooked the effect of island size. The Subsidized Island Biogeography Hypothesis [21] and other studies suggest that size can affect the impact of marine subsidy effects on animal populations, increasing species richness [21,78], population density [16,79] population abundance [10], and nitrogen enrichment [14]. Future studies with more precise sampling designs along transects from the coastline to the interior of different islands would provide better insights into the relationship between island size and marine subsidies in all lizard diets.

Finally, we expected a larger difference in isotopic values between insectivorous and herbivorous lizards and higher δ ^15^N values in insectivorous lizards. However, despite their distinct trophic positions, both groups shared a significant portion (almost 70%) of the isotopic space across the sampled islands. Both lizard groups showed relatively low variation in carbon isotopes, with less variation observed in iguanas, but they showed greater variation in nitrogen isotopes. This variation is likely to be due to differences in nitrogen sources between islands with and without significant seabird colonies (Figs 3 and 4) regardless of aridity.

This suggests that both lizard groups incorporate marine subsidies into their diets to a substantial degree. These findings are consistent with Obrist’s study [14], which emphasizes that the primary mechanism for marine nutrient transfer within island food webs is plant fertilization, rather than direct consumption of marine subsidies.

## Conclusion

Our hypothesis was that the size of the island, its degree of aridity or the presence of seabird colonies will be related to the amount of the marine food available for the lizards.

Overall, our study provides evidence for the role played by marine subsidies in the diets of both insectivorous and herbivorous lizards across the islands in the Gulf of California. We found higher nitrogen values and less negative carbon values in lizards inhabiting islands with significant seabird colonies and with hyper-arid conditions, regardless of the lizards’ foraging strategies. The ubiquity of seabird colonies combined with higher aridity in the northern part of the Gulf, creates a northward gradient in the importance of marine subsidies for both insectivorous and herbivorous lizards, across the Gulf islands as a whole.

We could not find a relationship with the size of the islands, but we suspect the design of our sampling was not adequate for it. Future research could focus on more detailed sampling along coastal-inland transects to better understand the spatial distribution of marine subsidies on islands of varying sizes. Additional isotopic analysis of plants and arthropods could provide a more comprehensive view of the nutrient flow within these island ecosystems.

## Supporting information

S1 Fig Isotopic values of insectivorous lizards and average isotopic data of arthropods. δ13C and δ15N values of insectivorous lizards (Uta stansburiana) from islands of the Gulf of California. The color pattern of each island corresponds to the aridity/latitudinal gradient shown in Figure 1. Averages and standard deviations of spiders (circles), scorpions (squares), carrion insects (triangles), and seabird ectoparasites (rhombus) data from Anderson and Polis (1998) (purple), herbivore insects (downward triangles), detritivores (dodecagons), predator insects (stars), and littoral invertebrates (hexagons) data from Stapp and Polis (2003) (pink), arthropods (crosses) data Barrett et al. (2005) (orange) are shown. Empty symbols represent habitats with the presence of seabirds, filled symbols represent non-seabird habitats, and dashed-line figures represent mainland. For more details regarding arthropods species or locality, see S2 Table.
(TIF)

S2 Fig Isotopic values of herbivorous lizards and average isotopic data of plants. δ13C and δ15N values of herbivorous iguanas from Mejia, San Esteban, Coronados and La Islita Islands. The color pattern of each island corresponds to the aridity/latitudinal gradient shown in Figure 1. Circles represent species of the *Sauromalus* genus, while squares represent *Dipsosaurus dorsalis*. Averages and standard deviations of C3 (circles), C4 (squares), and CAM (triangles) plants, as well as algae (rhombus) collected during our surveys (dark green), algae data from Anderson and Polis (1998) (purple), C3, C4/CAM plants, and algae data from Stapp and Polis (2003) (pink), C3, C4 plants, and algae data from Barrett et al. (2005) (orange), and C3, C4, and CAM plants data from Delibes et al. (2015) (black) are shown. Empty symbols represent habitats with the presence of seabirds, filled symbols represent non-seabird habitats, and dashed-line figures represent mainland. For more details regarding plant species or locality, see S1 Table.
(TIF)

S1 Table Average isotopic data of C3, C4, and CAM plants from our study and literature. Average and standard deviation of δ13C and δ15N of C3, C4, and CAM plants, as well as marine algae collected for this study on Angel de la Guarda Island. Average and standard deviation of δ13C and δ15N of C3, C4, and CAM plants, as well as marine algae found in the literature from some islands and coastal areas of the Gulf of California, as well as the Baja California Peninsula (mainland).
(DOCX)

S2 Table Average isotopic data of arthropods from the literature. Average and standard deviation of δ13C and δ15N of terrestrial and littoral arthropods found in the literature from some islands and coastal areas of the Gulf of California, as well as the Baja California Peninsula (mainland).
(DOCX)

S3 Table Raw isotopic data of insular lizards used in this study. δ13C ‰ and δ15N ‰ of tail-tissues of insectivorous and herbivorous lizards from different islands of the Gulf of California. Sampling year and C:N ratios are shown per individual. In shade the sampling period that could reflect effects of El Niño event.
(DOCX)
