# Supplementary figures and images for: Understanding the influence of marine nutrients on insectivorous and herbivorous reptiles in the Gulf of California islands
Source: PLoS One. 2025 Aug 22;20(8):e0329414. doi: 10.1371/journal.pone.0329414 (PMC12373247; doi:10.1371/journal.pone.0329414)

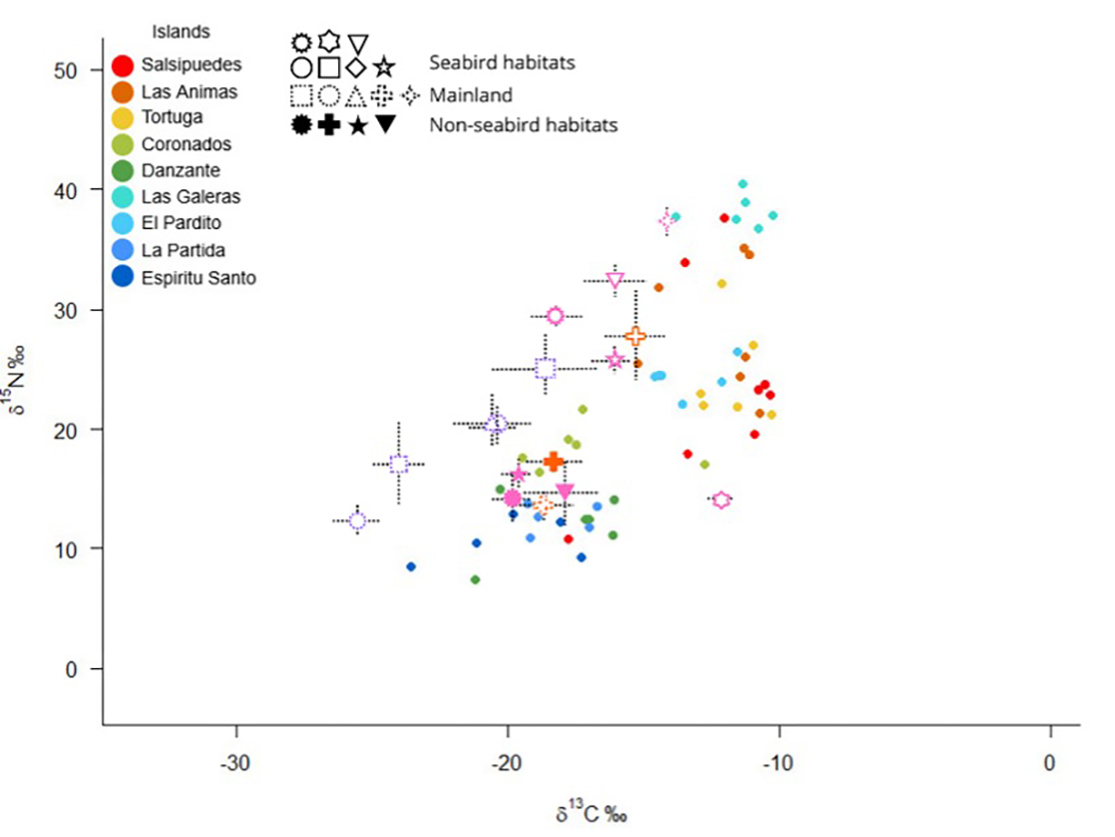

Supplement: S1 Fig — δ13C and δ15N values of insectivorous lizards (Uta stansburiana) from islands of the Gulf of California. The color pattern of each island corresponds to the aridity/latitudinal gradient shown in Figure 1. Averages and standard deviations of spiders (circles), scorpions (squares), carrion insects (triangles), and seabird ectoparasites (rhombus) data from Anderson and Polis (1998) (purple), herbivore insects (downward triangles), detritivores (dodecagons), predator insects (stars), and littoral invertebrates (hexagons) data from Stapp and Polis (2003) (pink), arthropods (crosses) data Barrett et al. (2005) (orange) are shown. Empty symbols represent habitats with the presence of seabirds, filled symbols represent non-seabird habitats, and dashed-line figures represent mainland. For more details regarding arthropods species or locality, see S2 Table. (TIF) [file pone.0329414.s001.tif]

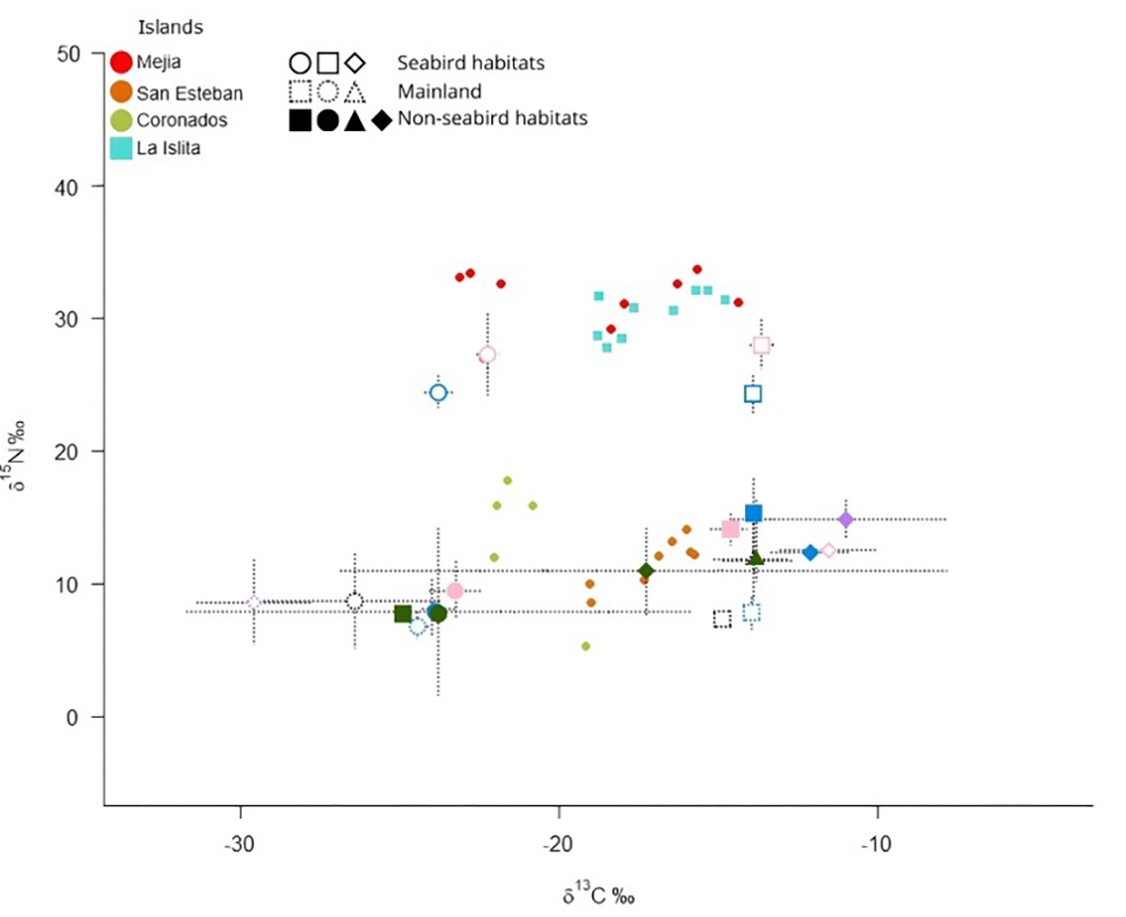

Supplement: S2 Fig — δ13C and δ15N values of herbivorous iguanas from Mejia, San Esteban, Coronados and La Islita Islands. The color pattern of each island corresponds to the aridity/latitudinal gradient shown in Figure 1. Circles represent species of the Sauromalus genus, while squares represent Dipsosaurus dorsalis. Averages and standard deviations of C3 (circles), C4 (squares), and CAM (triangles) plants, as well as algae (rhombus) collected during our surveys (dark green), algae data from Anderson and Polis (1998) (purple), C3, C4/CAM plants, and algae data from Stapp and Polis (2003) (pink), C3, C4 plants, and algae data from Barrett et al. (2005) (orange), and C3, C4, and CAM plants data from Delibes et al. (2015) (black) are shown. Empty symbols represent habitats with the presence of seabirds, filled symbols represent non-seabird habitats, and dashed-line figures represent mainland. For more details regarding plant species or locality, see S1 Table. (TIF) [file pone.0329414.s002.tif]
