# Supplementary material for: Understanding the influence of marine nutrients on insectivorous and herbivorous reptiles in the Gulf of California islands
Source: PLoS One. 2025 Aug 22;20(8):e0329414. doi: 10.1371/journal.pone.0329414 (PMC12373247; doi:10.1371/journal.pone.0329414)
Supplement: S1 Table — Average and standard deviation of δ13C and δ15N of C3, C4, and CAM plants, as well as marine algae collected for this study on Angel de la Guarda Island. Average and standard deviation of δ13C and δ15N of C3, C4, and CAM plants, as well as marine algae found in the literature from some islands and coastal areas of the Gulf of California, as well as the Baja California Peninsula (mainland). (DOCX) [file pone.0329414.s003.docx]

**S1 Table**. Average and standard deviation of δ^13^C and δ^15^N of C3, C4, and CAM plants, as well as marine algae collected for this study on Angel de la Guarda Island. Average and standard deviation of δ^13^C and δ^15^N of C3, C4, and CAM plants, as well as marine algae found in the literature from some islands and coastal areas of the Gulf of California, as well as the Baja California Peninsula (mainland)

| Plant type | Sampling area | Seabird presence | δ^13^C‰ ± SD | δ^15^N‰ ± SD | *n* | Family or species name | Source |
| --- | --- | --- | --- | --- | --- | --- | --- |
| C3 | Angel de la Guarda Island | No | -23.7±7.9 | 7.8±6.2 | 6 | *Larrea tridentata, Datura spp, Euphorbia lomelii, Marina spp., Batis marítima, Lupinus spp.* | This study |
| C3 | Islands of Bahia de los Angeles, Gulf of California | Yes | -22.2±0.3 | 27.2±3.0 | 5 | Amaranthaceae, Asteraceae, Onagraceae, Scrophulariaceae, Solanaceae, Zygophyllaceae | Stapp and Polis (2003) |
| C3 | Islands of Bahia de los Angeles, Gulf of California | No | -23.2±0.8 | 9.5±2.0 | 9 | Amaranthaceae, Asteraceae, Onagraceae, Scrophulariaceae, Solanaceae, Zygophyllaceae | Stapp and Polis (2003) |
| C3 | Piojo Island/ inland | Yes | -23.8±0.4 | 24.5±1.1 | 12 | - | Barrett et al (2005) |
| C3 | Bota, Mitlan, Pata, Smith, Ventana, Cabeza de Caballo Islands/ Coastal areas | No | -23.9±0.6 | 8.2±2.1 | 4 | - | Barrett et al (2005) |
| C3 | Mainland | No | -24.4±0.3 | 6.8±1.1 | 6 | - | Barrett et al (2005) |
| C3 | Southermost quarter of the Baja California Peninsula | Unspecified | -26.4±2.7 | 8.6±3.6 | 21 | Bignociaceae, Solanaceae, Fabaceae, Celastraceae, Zygophyllaceae, Euphorbiaceae, Fouquieriaceae, Asteraceae, Anacardiaceae, Burseraceae | Delibes et al (2015) |
| C3 | Angel de la Guarda Island | No | -24.8 | 7.8 | 1 | *Cucurbita cordata* | This study |
| C4/CAM | Islands of Bahia de los Angeles, Gulf of California | Yes | -13.6±0.3 | 28.0±1.8 | 12 | Aizoaceae, Cactaceae, Chenopodiaceae | Stapp and Polis (2003) |
| C4/CAM | Islands of Bahia de los Angeles, Gulf of California | No | -14.6±0.6 | 14.0±1.1 | 10 | Aizoaceae, Cactaceae, Chenopodiaceae | Stapp and Polis (2003) |
| C4 | Piojo Island/ inland | Yes | -13.9±0.2 | 24.3±1.4 | 11 | - | Barrett et al (2005) |
| C4 | Bota, Mitlan, Pata, Smith, Ventana, Cabeza de Caballo Islands/ Coastal areas | No | -13.9±0.1 | 14.5±3.5 | 5 | - | Barrett et al (2005) |
| C4 | Mainland | No | -14.0±0.2 | 7.8±1.2 | 7 | - | Barrett et al (2005) |
| C4 | Southermost quarter of the Baja California Peninsula | Unspecified | -14.8 | 7.3 | 1 | Gramineae | Delibes et al (2015) |
| CAM | Angel de la Guarda Island | No | -13.8±1.0 | 11.7±2.0 | 10 | *Stenocereus gummosus, Cylindropuntia cholla, C. imbricata, Pachycereus pringlei, Lophocereus schottii, Stenocereus thurberi, Mammillaria dioica, Opuntia fulgida, Agave spp., Echinocactus platycathus* | This study |
| CAM | Southermost quarter of the Baja California Peninsula | Unspecified | -13.9±1.2 | 11.8±3.1 | 25 | Cactaceae, Euphorbiaceae, Asparagaceae | Delibes et al (2015) |
| CAM | San Pedro Martir Island | Yes | - | 30.3±3.86 | 10 | *Pachycereus pringlei* | Wilder et al (2022) * |
| CAM | Partida, Cardonosa, Rasa, Salsipuedes, Las Animas, Cholludo, Alcatraz Islands | Yes | - | 30.3±3.44 | 10 | *Pachycereus pringlei* | Wilder et al (2022) * |
| CAM | San Lorenzo, San Esteban, Datil Islands | Yes | - | 11.7±2.26 | 10 | *Pachycereus pringlei* | Wilder et al (2022) * |
| CAM | Baja California Peninsula | Unspecified | - | 8.1±3.12 | 10 | - | Wilder et al (2022) * |
| Marine algae | Angel de la Guarda Island | No | -17.3±9.5 | 10.94±3.3 | 4 | - | This study |
| Marine algae | Bahia de los Angeles, Gulf of California/ Costal areas | Unspecified | -11.0±3.18 | 14.86±1.35 | 3 | - | Anderson and Polis (1998) |
| Marine algae | Bahia de los Angeles, Gulf of California/ Inland areas | Unspecified | -29.6±1.7 | 8.5±3.2 | 2 | - | Anderson and Polis (1998) |
| Marine algae | Islands of Bahia de los Angeles, Gulf of California | Yes | -11.5±1.5 | 12.5±0.4 | 7 | Sargassaceae, Ulvaceae | Stapp and Polis (2003) |
| Marine algae | Bota, Mitlan, Pata, Smith, Ventana, Cabeza de Caballo Islands/ Coastal areas | No | -12.1±1.2 | 12.4±0.5 | 9 | - | Barrett et al (2005) |
| Marine algae | Continental and peninsula Gulf of California coastlines | Unspecified | -12.55±3.07 | - | 241 | *Colpomenia spp., Padina spp., Sargassum spp.* | Soto-Jimenez et al (2022) * |
| Marine algae | Continental and peninsula Gulf of California coastlines | Unspecified | -14.5±3.0 | - | 174 | *Chaetomorpha spp., Codium spp., Ulva spp.* | Soto-Jimenez et al (2022) * |
| Marine algae | Continental and peninsula Gulf of California coastlines | Unspecified | -14.84±3.9 | - | 147 | *Gracilaria spp., Hypnea spp., Laurencia spp., Spyrida spp.* | Soto-Jimenez et al (2022) * |

*Not used for the ellipses’ analysis
