# Supplementary material for: Understanding the influence of marine nutrients on insectivorous and herbivorous reptiles in the Gulf of California islands
Source: PLoS One. 2025 Aug 22;20(8):e0329414. doi: 10.1371/journal.pone.0329414 (PMC12373247; doi:10.1371/journal.pone.0329414)
Supplement: S2 Table — Average and standard deviation of δ13C and δ15N of terrestrial and littoral arthropods found in the literature from some islands and coastal areas of the Gulf of California, as well as the Baja California Peninsula (mainland). (DOCX) [file pone.0329414.s004.docx]

**S2 Table**. Average and standard deviation of δ^13^C and δ^15^N of terrestrial and littoral arthropods found in the literature from some islands and coastal areas of the Gulf of California, as well as the Baja California Peninsula (mainland).

| Prey type | Sampling area | Seabird presence | δ^13^C‰ ± SD | δ^15^N‰ ± SD | *n* | Family or species name | Source |
| --- | --- | --- | --- | --- | --- | --- | --- |
| Spiders | Bahia de los Angeles, Gulf of California/ Costal areas | Unspecified | -20.4±0.8 | 20.2±1.6 | 17 | *-* | Anderson and Polis (1998) |
| Spiders | Bahia de los Angeles, Gulf of California/ Inland areas | Unspecified | -25.5±0.8 | 12.3±1.1 | 8 | - | Anderson and Polis (1998) |
| Carrion insects | Bahia de los Angeles, Gulf of California/ Coastal areas | Unspecified | -20.5±1.3 | 20.5±1.9 | 9 | - | Anderson and Polis (1998) |
| Seabird ectoparasites | Bahia de los Angeles, Gulf of California/ Coastal areas | Unspecified | -14.1±0.09 | 37.32±1.0 | 2 | - | Anderson and Polis (1998) |
| Scorpions | Bahia de los Angeles, Gulf of California/ Coastal areas | Unspecified | -18.6±1.9 | 25.2±2.4 | 4 | - | Anderson and Polis (1998) |
| Scorpions | Bahia de los Angeles, Gulf of California/ Inland areas | Unspecified | -24.0±0.9 | 17.0±3.4 | 5 | - | Anderson and Polis (1998) |
| Herbivores insects | Coronadito, Piojo, Flecha, and Blanca Islands, Gulf of California | Yes | -16.0±1.11 | 32.37±1.3 | 11 | Acrididae, Anobiidae, Curculionidae, Ptinidae, Psycidae | Stapp and Polis (2003) |
| Herbivores insects | Smith, Mitlán, Pata, Bota, Ventana, and Cabeza de Caballo Islands, Gulf of California | No | -18.0±1.3 | 14.6±2.6 | 10 | Acrididae, Anobiidae, Curculionidae, Ptinidae, Psycidae | Stapp and Polis (2003) |
| Detritivores  insects | Coronadito, Piojo, Flecha, and Blanca Islands, Gulf of California | Yes | -18.2±0.9 | 29.3±0.7 | 13 | Lepismatidae, Tenebrionidae | Stapp and Polis (2003) |
| Detritivores  insects | Smith, Mitlán, Pata, Bota, Ventana, and Cabeza de Caballo Islands, Gulf of California | No | -19.85±0.7 | 14.13±1.9 | 15 | Lepismatidae, Tenebrionidae | Stapp and Polis (2003) |
| Predators arthropods | Coronadito, Piojo, Flecha, and Blanca Islands, Gulf of California | Yes | -16.1±0.75 | 25.7±1.0 | 13 | Agelenidae, Araenidae, Lycosidae | Stapp and Polis (2003) |
| Predators arthropods | Smith, Mitlán, Pata, Bota, Ventana, and Cabeza de Caballo Islands, Gulf of California | No | -19.6±0.4 | 16.1±1.1 | 6 | Agelenidae, Araenidae, Lycosidae | Stapp and Polis (2003) |
| Littoral invertebrates | Coronadito, Piojo, Flecha, and Blanca Islands, Gulf of California | Yes | -12.1±0.4 | 14.1±0.4 | 34 | Muricidae, Cancridae, Diogenidae, Gammaridae, Ligiidae, Tenebrionidae, Histeridae | Stapp and Polis (2003) |
| Arthropods | Piojo Island/ inland | Yes | -15.3±1.1 | 27.8±3.6 | 7 | - | Barrett et al (2005) |
| Arthropods | Bota, Mitlan, Pata, Smith, Ventana, Cabeza de Caballo Islands/ Coastal areas | No | -18.3±1.0 | 17.3±0.7 | 25 | - | Barrett et al (2005) |
| Arthropods | Mainland | No | -18.7±1.0 | 13.6±1.1 | 9 | - | Barrett et al (2005) |
