# Supplementary material for: Understanding the influence of marine nutrients on insectivorous and herbivorous reptiles in the Gulf of California islands
Source: PLoS One. 2025 Aug 22;20(8):e0329414. doi: 10.1371/journal.pone.0329414 (PMC12373247; doi:10.1371/journal.pone.0329414)
Supplement: S3 Table — δ13C ‰ and δ15N ‰ of tail-tissues of insectivorous and herbivorous lizards from different islands of the Gulf of California. Sampling year and C:N ratios are shown per individual. In shade the sampling period that could reflect effects of El Niño event. (DOCX) [file pone.0329414.s005.docx]

**S3 Table**. δ^13^C ‰ and δ^15^N ‰ of tail-tissues of insectivorous and herbivorous lizards from different islands of the Gulf of California. Sampling year and C:N ratio are shown per individual. In shade the sampling period that could reflect effects of El Niño event.

|  | Island | Sampling period | δ^13^C ‰ (V-PDB) | δ^15^N ‰ (Air-N_2_) | C: N Ratio |
| --- | --- | --- | --- | --- | --- |
| **Insectivorous lizards** | | | | | |
| *U. stansburiana* | Angel de la Guarda | Sept 2014 | -15.71 | 13.52 | 3.7 |
| *U. stansburiana* | Salsipuedes | Sept 2014 | -13.38 | 17.94 | 3.2 |
| *U. stansburiana* | Salsipuedes | Sept 2014 | -10.37 | 22.86 | 3.3 |
| *U. stansburiana* | Salsipuedes | Sept 2014 | -10.94 | 19.55 | 3.5 |
| *U. stansburiana* | Salsipuedes | Sept 2014 | -10.79 | 23.30 | 3.3 |
| *U. stansburiana* | Salsipuedes | Sept 2014 | -10.53 | 23.76 | 3.8 |
| *U. stansburiana* | Salsipuedes | Sept 2014 | -13.49 | 33.93 | 3.6 |
| *U. stansburiana* | Salsipuedes | Sept 2014 | -17.81 | 10.84 | 3.7 |
| *U. stansburiana* | Salsipuedes | Nov 2013 | -12.02 | 37.68 | 3.9 |
| *U. stansburiana* | Las Animas | Sept 2014 | -11.47 | 24.44 | 3.7 |
| *U. stansburiana* | Las Animas | Sept 2014 | -15.21 | 25.49 | 4.3 |
| *U. stansburiana* | Las Animas | Sept 2014 | -10.72 | 21.31 | 3.6 |
| *U. stansburiana* | Las Animas | Sept 2014 | -11.26 | 25.99 | 3.6 |
| *U. stansburiana* | Las Animas | May 2015 | -11.10 | 34.52 | 3.4 |
| *U. stansburiana* | Las Animas | May 2015 | -11.33 | 35.09 | 3.6 |
| *U. stansburiana* | Las Animas | May 2015 | -14.48 | 31.81 | 4.6 |
| *U. stansburiana* | San Esteban | Sept 2014 | -12.63 | 15.07 | 3.8 |
| *U. stansburiana* | San Esteban | Sept 2014 | -13.81 | 14.19 | 3.7 |
| *U. stansburiana* | San Esteban | Sept 2014 | -14.30 | 13.07 | 3.5 |
| *U. stansburiana* | San Esteban | Sept 2014 | -13.03 | 14.34 | 3.5 |
| *U. stansburiana* | San Lorenzo | Nov 2013 | -12.95 | 23.21 | 3.6 |
| *U. palmeri* | San Pedro Martir | Nov 2013 | -12.72 | 24.97 | 3.6 |
| *U. palmeri* | San Pedro Martir | Nov 2013 | -13.94 | 25.82 | 3.7 |
| *U. palmeri* | San Pedro Martir | Nov 2013 | -12.98 | 24.65 | 3.6 |
| *U. palmeri* | San Pedro Martir | Nov 2013 | -14.05 | 20.68 | 3.6 |
| *U. stansburiana* | Tortuga | Oct 2014 | -12.14 | 32.13 | 3.8 |
| *U. stansburiana* | Tortuga | Oct 2014 | -12.80 | 21.96 | 3.9 |
| *U. stansburiana* | Tortuga | Oct 2014 | -10.97 | 27.05 | 3.6 |
| *U. stansburiana* | Tortuga | Oct 2014 | -11.57 | 21.91 | 3.6 |
| *U. stansburiana* | Tortuga | Oct 2014 | -10.30 | 21.18 | 4.2 |
| *U. stansburiana* | Tortuga | Oct 2014 | -12.91 | 22.98 | 3.9 |
| *U. stansburiana* | Coronados | Apr 2018 | -17.28 | 21.69 | 3.5 |
| *U. stansburiana* | Coronados | Apr 2018 | -17.78 | 19.09 | 3.5 |
| *U. stansburiana* | Coronados | Apr 2018 | -19.49 | 17.62 | 3.5 |
| *U. stansburiana* | Coronados | Apr 2018 | -18.86 | 16.40 | 3.5 |
| *U. stansburiana* | Coronados | Apr 2018 | -17.51 | 18.72 | 3.5 |
| *U. stansburiana* | Coronados | Apr 2018 | -12.78 | 17.02 | 3.5 |
| *U. stansburiana* | Danzante | Sept 2021 | -17.02 | 12.48 | 3.5 |
| *U. stansburiana* | Danzante | Sept 2021 | -20.32 | 14.99 | 4.5 |
| *U. stansburiana* | Danzante | Sept 2021 | -16.16 | 11.10 | 3.6 |
| *U. stansburiana* | Danzante | Sept 2021 | -17.15 | 12.41 | 3.4 |
| *U. stansburiana* | Danzante | Sept 2021 | -16.11 | 14.09 | 3.4 |
| *U. stansburiana* | Danzante | Sept 2021 | -21.21 | 7.44 | 2.5 |
| *U. stansburiana* | Las Galeras | Sept 2021 | -11.25 | 38.96 | 3.6 |
| *U. stansburiana* | Las Galeras | Sept 2021 | -10.27 | 37.84 | 3.5 |
| *U. stansburiana* | Las Galeras | Sept 2021 | -13.82 | 37.78 | 4.3 |
| *U. stansburiana* | Las Galeras | Sept 2021 | -11.36 | 40.48 | 3.6 |
| *U. stansburiana* | Las Galeras | Sept 2021 | -10.76 | 36.79 | 3.4 |
| *U. stansburiana* | Las Galeras | Sept 2021 | -11.59 | 37.49 | 3.5 |
| *U. squamata* | Santa Catalina | May 2018 | -18.71 | 14.48 | 3.9 |
| *U. stansburiana* | El Pardito | Feb 2020 | -12.15 | 23.95 | 3.4 |
| *U. stansburiana* | El Pardito | Feb 2020 | -13.59 | 22.12 | 3.2 |
| *U. stansburiana* | El Pardito | Feb 2020 | -14.44 | 24.46 | 4.0 |
| *U. stansburiana* | El Pardito | Feb 2020 | -14.61 | 24.38 | 4.1 |
| *U. stansburiana* | El Pardito | Feb 2020 | -11.55 | 26.49 | 3.3 |
| *U. stansburiana* | La Partida | Jun 2015 | -23.60 | 8.55 | 6.8 |
| *U. stansburiana* | La Partida | Jun 2015 | -19.84 | 12.90 | 4.4 |
| *U. stansburiana* | La Partida | Jun 2015 | -17.33 | 9.24 | 3.2 |
| *U. stansburiana* | La Partida | Jun 2015 | -18.06 | 12.20 | 3.6 |
| *U. stansburiana* | La Partida | Jun 2015 | -21.16 | 10.48 | 4.4 |
| *U. stansburiana* | Espiritu Santo | Oct 2015 | -18.90 | 12.63 | 3.8 |
| *U. stansburiana* | Espiritu Santo | Oct 2015 | -19.30 | 13.79 | 3.6 |
| *U. stansburiana* | Espiritu Santo | Oct 2015 | -16.72 | 13.50 | 3.7 |
| *U. stansburiana* | Espiritu Santo | Oct 2015 | -19.18 | 10.91 | 3.7 |
| *U. stansburiana* | Espiritu Santo | Oct 2015 | -17.03 | 11.83 | 3.7 |
| **Herbivorous lizards** | | | | | |
| *Sauromalus hispidus* | Mejia | May 2015 | -17.94 | 31.10 | 3.8 |
| *S. hispidus* | Mejia | May 2015 | -16.31 | 32.64 | 4.2 |
| *S. hispidus* | Mejia | May 2015 | -15.66 | 33.75 | 3.7 |
| *S. hispidus* | Mejia | May 2015 | -18.36 | 29.22 | 3.9 |
| *S. hispidus* | Mejia | May 2015 | -23.15 | 33.14 | 4.1 |
| *S. hispidus* | Mejia | May 2015 | -14.38 | 31.19 | 3.8 |
| *S. hispidus* | Mejia | May 2015 | -21.82 | 32.59 | 3.8 |
| *S. hispidus* | Mejia | May 2015 | -22.37 | 26.97 | 4.4 |
| *S. hispidus* | Mejia | May 2015 | -22.81 | 33.40 | 4.4 |
| *S. hispidus* | Angel de la Guarda | May 2015 | -22.96 | 2.01 | 3.8 |
| *S. varius* | San Esteban | May 2015 | -16.44 | 13.18 | 4.2 |
| *S. varius* | San Esteban | May 2015 | -15.75 | 12.15 | 3.6 |
| *S. varius* | San Esteban | May 2015 | -16.00 | 14.08 | 4.0 |
| *S. varius* | San Esteban | May 2015 | -15.86 | 12.38 | 3.9 |
| *S. varius* | San Esteban | May 2015 | -16.89 | 12.07 | 3.8 |
| *S. varius* | San Esteban | May 2015 | -20.39 | 10.77 | 3.7 |
| *S. varius* | San Esteban | May 2015 | -19.05 | 10.00 | 3.8 |
| *S. varius* | San Esteban | May 2015 | -17.34 | 10.28 | 3.8 |
| *S. varius* | San Esteban | May 2015 | -19.02 | 8.62 | 3.7 |
| *Ctenosaura conspicuosa* | San Esteban | Sept 2014 | -16.79 | 11.48 | 3.7 |
| *C. conspicuosa* | San Esteban | Sept 2014 | -15.00 | 13.10 | 3.7 |
| *C. conspicuosa* | San Esteban | Sept 2014 | -18.48 | 7.65 | 4.1 |
| *S. slevini* | Coronados | Apr 2018 | -19.16 | 5.25 | 3.8 |
| *S. slevini* | Coronados | Apr 2018 | -21.95 | 15.89 | 3.7 |
| *S. slevini* | Coronados | Sept 2021 | -21.62 | 17.76 | 4.0 |
| *S. slevini* | Coronados | Sept 2021 | -22.04 | 11.95 | 3.6 |
| *S. slevini* | Coronados | Sept 2021 | -20.83 | 15.86 | 3.7 |
| *Dipsosaurus dorsalis* | Coronados | Sept 2014 | -22.88 | 10.18 | 4.1 |
| *D. dorsalis* | Coronados | Sept 2021 | -22.31 | 9.81 | 3.6 |
| *D. dorsalis* | Coronados | Sept 2021 | -25.92 | 8.34 | 3.5 |
| *S. obesus* | Danzante | Sept 2021 | -19.65 | 8.99 | 3.6 |
| *S. obesus* | Danzante | Sept 2021 | -17.10 | 7.59 | 3.4 |
| *D. dorsalis* | La Islita | Sept 2021 | -17.67 | 30.80 | 4.5 |
| *D. dorsalis* | La Islita | Sept 2021 | -18.80 | 28.65 | 4.9 |
| *D. dorsalis* | La Islita | Sept 2021 | -16.40 | 30.55 | 4.3 |
| *D. dorsalis* | La Islita | Sept 2021 | -15.69 | 32.12 | 3.8 |
| *D. dorsalis* | La Islita | Sept 2021 | -18.03 | 28.51 | 3.6 |
| *D. dorsalis* | La Islita | Sept 2021 | -18.49 | 27.84 | 3.9 |
| *D. dorsalis* | La Islita | Sept 2021 | -18.76 | 31.66 | 4.1 |
| *D. dorsalis* | La Islita | Sept 2021 | -15.32 | 32.09 | 3.6 |
| *D. dorsalis* | La Islita | Sept 2021 | -14.78 | 31.41 | 3.7 |
| *D. catalinensis* | Santa Catalina | May 2018 | -14.59 | 13.60 | 3.6 |
| *S. obesus* | Espiritu Santo | Apr 2015 | -22.69 | 4.68 | 3.9 |
| *S. obesus* | Espiritu Santo | Apr 2015 | -19.77 | 10.65 | 3.6 |
